# Improved intention, self-efficacy and social influence in the workspace may help low vision service workers to discuss depression and anxiety with visually impaired and blind adults

**DOI:** 10.1186/s12913-022-07944-0

**Published:** 2022-04-21

**Authors:** Edine P. J. van Munster, Hilde P. A. van der Aa, Peter Verstraten, Martijn W. Heymans, Ruth M. A. van Nispen

**Affiliations:** 1 Amsterdam UMC location Vrije Universiteit Amsterdam, Ophthalmology, De Boelelaan 1117, Amsterdam, the Netherlands; 2 Amsterdam Public Health, Quality of Care, Mental Health, Aging and Later Life, Amsterdam, the Netherlands; 3 Robert Coppes Foundation, Expertise, Innovation and Knowledge, Vlasmeersestraat 38-A, Vught, the Netherlands; 4 The Lighthouse Guild NYC, 250 W 64th St, New York, USA; 5 Amsterdam UMC location Vrije Universiteit Amsterdam, Epidemiology and Data Science, De Boelelaan 1117, Amsterdam, the Netherlands

**Keywords:** Low vision, Vision impairment, Depression, Anxiety, Detection, Professional development

## Abstract

**Background:**

Depression and anxiety are common in visually impaired and blind adults, but often remain untreated in those who receive support from low vision service (LVS) organizations. This study aims to determine factors associated with discussing mental health by LVS workers.

**Methods:**

A self-administered cross-sectional survey in one hundred LVS workers was performed. Data on current practice, symptom attribution, and determinants of the Integrated Change Model (i.e. predisposing and environmental factors, awareness, attitude, self-efficacy, social influence, confidence and barriers) were investigated. Multivariable logistic regression analysis was performed to determine predictors of discussing mental health problems in this population. Subsequently, internal validation was conducted using a bootstrapping method.

**Results:**

Around 80% of the participants often discussed mental health with clients. Five factors were found to predict discussion of mental health: female gender (OR = 4.51; 95% confidence interval (CI) 0.98 to 21.61), higher education (OR = 3.39; CI 1.19 to 9.66), intention to discuss mental health problems (OR = 3.49; CI 1.20 to 10.15), higher self-efficacy (OR = 1.11; CI 1.02 to 1.20), and higher perceived social influence (OR = 1.15; CI 1.05 to 1.27). Good discrimination after internal validation was reflected by the area under the curve (0.850).

**Conclusions:**

Previous studies indicate clients want healthcare providers to initiate discussions about mental health. However, still 20% of LVS workers do not discuss suspected depression or anxiety. In order to improve this, LVS organizations could address mental health as part of their care and provide training to ensure intention to discuss mental health problems, improve self-efficacy and create a supportive environment between colleagues.

**Supplementary Information:**

The online version contains supplementary material available at 10.1186/s12913-022-07944-0.

## Background

More than half of the visually impaired and blind older adults who experience (subthreshold) depression or anxiety lack professional mental health support [1, 2], and depression and anxiety often go undetected [1–3]. This is concerning, since about one in three middle aged and older adults with vision impairment (VI) or blindness experience subthreshold symptoms of depression or anxiety [2, 4, 5]. Working age adults report lower levels of mental health as well [6]. Moreover, in this population prevalence estimates of major depressive disorders range between 7% and 15.6% [7], compared to 4% to 5% in older adults [4, 7]. Early detection of mental health problems in all adults with VI offers the opportunity to intervene and prevent negative outcomes across all age groups. Without treatment, they are at high risk of developing a clinical depressive and/or anxiety disorder [4, 8]. Moreover, even symptoms of depression or anxiety can have a negative effect on quality of life and can decrease the visual and physical condition of individuals [9, 10].

Adults with VI report difficulties in identifying and discussing mental health problems. Previous studies show that clients from low vision service (LVS) organizations experience a lack of knowledge about mental health problems and possibilities for support, they often tend to focus on physical symptoms, and experience difficulties in distinguishing depression from normal grief due to vision loss [1, 11, 12]. Self-perception of having mental health problems varies among people with VI [12]. Mental health problems are often related to being visually impaired, and adults with VI feel the need to acknowledge their VI before they can initiate a conversation about depression or anxiety [11]. Moreover, they tend to rely on their own resources to deal with mental health problems and experience self-stigma related to their VI and mental health [1, 11, 12]. In their opinion, healthcare providers can have an important influence on early detection and discussion of mental health problems [11].

Healthcare providers who support adults with VI, experience difficulties in identifying and discussing mental health. Previous research in Wales and Australia showed that one in three rehabilitation workers and two in three eye care practitioners do not aim to detect symptoms of depression in their clients [13, 14]. Healthcare providers who felt less confident, perceived more barriers, and thought depression is a harmless and untreatable condition were less likely to attempt to detect depression [13, 14]. Most healthcare providers were positive towards receiving training in depression management [15].

Although these former studies have provided some insight, it remains largely unclear what factors encourage or prevent LVS workers to discuss mental health problems with their clients. Furthermore, previous studies primarily examined depression and less often focused on anxiety, even though anxiety in adults with VI is highly prevalent as well and is often comorbid with depression [4, 16]. Therefore, the aim of this study was to identify associated factors in LVS workers to discuss depression and anxiety in adults with VI. These insights can contribute to improving detection of mental health problems in this population, and subsequently providing them adequate support.

## Methods

### Study design and participants

A cross-sectional study was conducted between April-September 2020. In the Netherlands, visually impaired and blind adults can receive specific vision-related support from three LVS organizations. This study was conducted in healthcare providers working at one of these three Dutch LVS organizations. Eligible participants were those working as an occupational therapist, a social worker, a counsellor (providing inpatient or outpatient care) or a professional who performs service eligibility assessments (assessors). These professionals were selected, because they are the first to get in contact with clients and are potentially able to detect mental health problems early on. Professionals were excluded from participation if they worked less than six months within low vision services. Contact persons from every organization purposively sent study invitation e-mails including an information letter and consent form to 352 eligible professionals. After providing digital consent, participants received a link to an online 30-min survey. To encourage participants to complete the survey, automatic reminders were sent after two weeks.

### Theoretical framework and questionnaire development

**Fig. 1:**
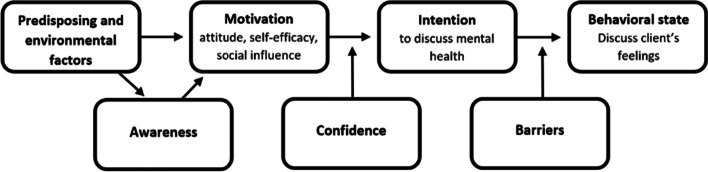
The Integrated Change (I-Change) model in which performance skills was replaced by confidence

The Integrated Change (I-Change) model was used as a theoretical framework [17], since the model can be used to examine determinants of health related behavior in professionals [18–20]. The I-Change model integrates several models on social cognitions [17]. It states that health behavior is determined by behavioral intention, in turn affected by motivational factors (i.e. attitude, self-efficacy and social influence). Knowledge, cues to action and risk perception (e.g. awareness) are determined by predisposing factors, and both predisposing factors and awareness influence motivational factors. Despite the fact that someone has the intention to show health related behavior, this can be affected by a lack of actual skills or perceived barriers. Since performance skills to discuss depression and anxiety were not directly measurable, it was replaced by healthcare providers’ confidence in depression and anxiety management, which was also done in previous research using the I-Change model [19]. Previous studies showed that healthcare provider’s confidence is related to their aim to identify depression [13, 14]. Therefore, confidence was relocated between motivation and intention (Fig. 1). Perceptions of adults with VI are also covered by use of the I-Change model, since they reported insufficient knowledge, attitude and skills in their healthcare providers as barriers in recognizing and discussing mental health problems [11].

The questionnaire used in this study was based on questionnaires from previous studies using the I-Change model, [18, 19] and studies on detection of depression by eye care practitioners and rehabilitation workers from Wales and Australia [13, 14]. Items were fitted within scales, adjusted for use in low vision studies if needed or removed when unapplicable. One researcher (EvM) translated the draft questionnaire into Dutch. To ensure a valid translation another researcher (HvdA) translated the questionnaire backwards to English. Thereafter, differences with the draft questionnaire were marked and discrepancies in translations were discussed to reach consensus. Subsequently, the draft questionnaire was piloted in three LVS workers to check comprehensibility and usability. See Additional file 1 for the final questionnaire.

### Main outcome measure

The main outcome measure was if LVS workers discussed depression and anxiety with their clients. Participants answered the question “If you suspect depression or anxiety in a client, how likely are you to discuss the client’s feelings?”, scoring on a 4-point Likert-scale, i.e. never, rarely, sometimes and often. Individual responses were dichotomized (never, rarely and sometimes = no, often = yes).

### Descriptive measures: symptoms and management strategies

Descriptive measures were assessed to determine participants’ attribution of symptoms of depression and anxiety, and use of depression and anxiety management strategies (Additional file 1, part 3). Symptoms were derived from the diagnostic criteria for major depressive disorder, generalized anxiety disorder, agoraphobia and social phobia [21]. These disorders are the most prevalent in adults with VI [4].

### Potential predictor variables

Predisposing and environmental factors were assessed (Additional file 1, part 1). Uneven distributions in educational level, profession and average contact frequency per client were found and therefore these were dichotomized. Intention to discuss mental health (but not actually doing so) was dichotomized by scoring a response of definitely as 1 and others as 0. Classical test theory was used by computing sum scores for each scale, i.e. awareness, attitude, self-efficacy, social influence, confidence and barriers, with higher scores indicating more of the underlying construct. Additional file 2 provides more details on psychometric measurements.

### Statistical analysis

All statistical analyses were conducted in R (version 4.0.3). Since participants were unable to finish the digital survey if a question remained unanswered, the sample was free of missings. Descriptive statistics were used to describe participant characteristics, symptom attribution and use of depression and anxiety management strategies. A correlation matrix was conducted to assess multicollinearity between potential predictors (*r* > 0.70), which was found between awareness of depression and anxiety (*r* = 0.85), and confidence in depression and anxiety management (*r* = 0.97). Therefore, anxiety and depression were assumed to be similar constructs in relation to awareness and confidence, and only depression was included as representative of mental health problems in the analyses. In addition, the linearity assumption was checked and when violated, restricted cubic splines were used with three knots located at the 10^th^, 50^th^ and 90^th^ percentile score of the variable [22]. This was the case for average clients per week and average time per consultation.

Univariable logistic regression analyses were performed to examine the relationship between LVS workers’ initiative to discuss mental health and all potential predictor variables. Multivariable logistic regression analyses using backward stepwise selection (*P* > 0.157 for removal of variables) was performed to predict discussion of mental health by LVS workers. A *P*-value of 0.157 was used, since a higher value should be considered in smaller datasets [23]. Performance of the final model was assessed by examining measures of overall performance and predictive performance (calibration and discrimination). Nagelkerke R2 and the Brier score were used as overall performance measures, where Nagelkerke R2 can be used to characterize the proportion of variation in the outcome variable explained by the model, and the Brier score calculates the disagreement between expected rates and the binary outcome variable. Calibration refers to the agreement between the model’s predictions and observed outcomes, and was examined by plotting predicted probabilities with the observed outcomes, and using the Hosmer–Lemeshow test. Discrimination refers to the prediction model’s ability to differentiate between those who discuss feelings and those who do not, and was examined with the area under the ROC curve (AUC).

Internal validity of the model was assessed with a bootstrapping procedure to determine realistic estimates of the regression coefficients and performance of the prediction model in LVS workers. The bootstrapping validation was performed in 1000 samples drawn with replacement from the original sample. This procedure provided estimates of optimism for performance measures. These estimates were subtracted from the values in the original dataset, which lead to optimism corrected R2, Brier score and AUC. In addition, bootstrapping provided a shrinkage factor that was used to correct for optimism in the regression coefficients by multiplying the original coefficients and the shrinkage factor. Adjusting for optimism is especially important in smaller sample sizes [24]. Subsequently, the recalibrated model’s calibration and discrimination were examined by a calibration plot, the Hosmer–Lemeshow test and AUC.

## Results

### Participant characteristics

One hundred LVS workers (13% male) participated in this study, which corresponds to a response rate of 28.4%. All participants thought that detection of depression and anxiety (mental health problems) is part of their job. On average participants were positive about discussing mental health problems with their clients (attitude) and experienced low levels of barriers, but also reported low scores on self-efficacy (Table 1).

**Table 1:** Participant characteristics (*n* = 100)

	**n (%)**
**Gender** (male)	13 (13)
**Educational level**	
Vocational training	26 (26)
Higher education or University	74 (74)
**Profession**	
Occupational therapist / assessor	19 (19)
Counsellor	61 (61)
Social worker	20 (20)
**Average contact frequency per client**	
< 10 times	41 (41)
> 10 times	59 (59)
**Detection is part of my job**	100 (100)
**Intention to discuss suspected symptoms** (yes)	56 (56)
**Discuss feelings** (yes)	81 (81)
	**Mean (SD)**^a^
**Age** in years	45.33 (11.00)
**Work experience in low vision practice** in years	12.68 (9.97)
**Average client contacts** per week	11.57 (8.44)
**Average time per consultation** in minutes	82.19 (44.80)
**Awareness (depression)** (scale 0–39)	26.54 (3.55)
**Awareness (anxiety)** (scale 0–39)	26.60 (3.36)
**Attitude** (scale 0–48)	38.32 (4.66)
**Self-efficacy** (scale 0–42)	19.43 (7.51)
**Social influence** (scale 0–42)	33.50 (5.37)
**Confidence (depression)** (scale 0–39)	22.52 (8.27)
**Confidence (anxiety)** (scale 0–39)	21.91 (7.91)
**Barriers** (scale 0–57)	20.44 (7.36)

*N* Number, *SD* Standard deviation

^a^medians were similar

### Symptoms and management strategies

Table 2 provides an overview of the distributions of participants’ responses on all 28 symptoms of depression and anxiety. Except for physical complaints in anxiety, participants mostly assigned symptoms to both VI and mental health. Depressed mood, loss of interest in activities, sleeping problems, fatigue, worth-, hope-, and helplessness, worrying about the future, loss of control, staying at home and avoiding (social) situations were more often attributed to both mental health problems and VI.

**Table 2:** Overview of participants’ attribution of symptoms of depression and anxiety

What do you think the following symptoms could be related to? Select by ticking a box for “VI”, “Depression “, “Both “ or “Neither “	**Symptom related to VI, *n* (%)**	**Symptom related to depression, *n *(%)**	**Symptom related to both depression and VI, *n* (%)**	**Symptom not related to either depression or VI, *n* (%)**
**Depressed mood**	4 (4%)	10 (10%)	86 (86%)	0 (0%)
**Loss of interest in activities**	2 (2%)	3 (3%)	94 (94%)	1 (1%)
**Sudden weight loss or increase**	0 (0%)	40 (40%)	58 (58%)	2 (2%)
**Decrease or increase of appetite **	0 (0%)	49 (49%)	51 (51%)	0 (0%)
**Sleeping problems**	3 (3%)	12 (12%)	85 (85%)	0 (0%)
**Fatigue**	4 (4%)	2 (2%)	94 (94%)	0 (0%)
**Worthlessness**	2 (2%)	6 (6%)	91 (91%)	1 (1%)
**Concentration problems**	5 (5%)	13 (13%)	82 (82%)	0 (0%)
**Recurring thoughts about death**	1 (1%)	44 (44%)	54 (54%)	1 (1%)
**Decreased interest in sex**	2 (2%)	46 (46%)	49 (49%)	3 (3%)
**Hopelessness**	1 (1%)	12 (12%)	87 (87%)	0 (0%)
**Irritation**	8 (8%)	10 (10%)	78 (78%)	4 (4%)
**Feelings of guilt**	10 (10%)	22 (22%)	57 (57%)	11 (11%)
**Physical symptoms e.g. heavy limbs, headaches, back pain and muscle pain**	6 (6%)	20 (22%)	64 (64%)	10 (10%)
**What do you think the following symptoms could be related to? Select by ticking a box for “VI”, “Anxiety “, “Both “ or “Neither “**	**Symptom related to VI, *n* (%)**	**Symptom related to anxiety, *n* (%)**	**Symptom related to both anxiety and VI, *n* (%)**	**Symptom not related to either anxiety or VI, *n* (%)**
**Restlessness**	2 (2%)	35 (35%)	61 (61%)	2 (2%)
**Fatigue**	22 (22%)	1 (1%)	77 (77%)	0 (0%)
**Concentration problems**	3 (3%)	14 (14%)	81 (81%)	2 (2%)
**Irritability**	5 (5%)	20 (20%)	73 (73%)	2 (2%)
**Sleeping problems**	2 (2%)	11 (11%)	87 (87%)	0 (0%)
**Worrying about the future**	3 (3%)	8 (8%)	89 (89%)	0 (0%)
**Ruminating**	1 (1%)	15 (15%)	84 (84%)	0 (0%)
**Helplessness**	10 (10%)	1 (1%)	89 (89%)	0 (0%)
**Loss of control**	8 (8%)	3 (3%)	89 (89%)	0 (0%)
**Avoiding (social) situations**	1 (1%)	4 (4%)	94 (94%)	1 (1%)
**Staying at home**	2 (2%)	4 (4%)	94 (94%)	0 (0%)
**Uncomfortable being alone**	2 (2%)	25 (25%)	71 (71%)	2 (2%)
**Muscle tensions**	2 (2%)	39 (39%)	58 (58%)	1 (1%)
**Physical symptoms e.g. shaking, hyperventilation and palpitations**	1 (1%)	73 (73%)	26 (26%)	0 (0%)

*VI* Vision impairment, *n* Number

Participants reported they most likely discussed client’s feelings, discussed their concerns about mental health problems with clients or colleagues, and reported concerns in a medical file whenever they suspected mental health problems. They less often provided written or verbal information, and 85% of the participants never used a questionnaire. Almost all participants discussed referral options regularly, preferably referrals to general practitioners and psychologists (Additional file 3).

### Prediction model

Results of the univariable and multivariable logistic regression analyses are shown in Table 3. Univariable logistic regression analyses showed that gender, educational level, intention to discuss, attitude, self-efficacy, social influence, confidence and barriers were related to the likelihood that healthcare providers discussed the client’s feelings. Gender, education, intention, self-efficacy and social influence were significant predictors of discussing mental health (*P* < 0.157). The odds of discussing feelings increased when participants were female (OR = 4.51, 95% Confidence Interval (CI): 0.98 to 21.61), had a higher education (OR = 5.07, 95% CI: 1.40 to 20.10), had the intention to discuss mental health problems (OR = 3.76, 95% CI: 1.10 to 14.83), reported higher self-efficacy (OR = 1.09, 95% CI: 1.00 to 1.21) and reported higher social influence within the LVS organization (OR = 1.15, 95% CI: 1.03 to 1.31).

**Table 3:** Overview of univariable and multivariable analyses predictors including internal validation and recalibration

	**Univariable logistic regression**			**Multivariable logistic regression**			**Multivariable logistic regression**^a^			**Recalibration of linear predictor**^b^	
**Predictor**	**β**	**OR (95% CI)**	***P***	**β**	**OR (95% CI)**	***P***	**β**	**OR (95% CI)**	***P***	**β**	**OR**
**Age**	-0.03	0.97 (0.92 to 1.02)	.23*								
**Gender** (female vs male)	1.58	4.88 (1.41 to 16.85)	.01*	1.51	4.51 (0.98 to 21.61)	.05	1.45	4.26 (0.93 to 19.47)	.06	1.06	2.88
**Profession** (occupational therapists and assessors vs social workers and counsellors)	-0.27	0.76 (0.20 to 2.94)	.69								
**Educational level** (higher education vs vocational training)	1.22	3.39 (1.19 to 9.66)	.02*	1.62	5.07 (1.40 to 20.10)	.02	1.59	4.89 (1.32 to 18.12)	.02	1.14	3.13
**Work experience low vision**	-0.01	0.99 (0.94 to 1.04)	.75								
**Average clients per week**											
Average clients per week	-0.10	0.91 (0.74 to 1.12)	.37								
Average clients per week^c^	0.21	1.23 (0.76 to 1.98)	.40								
**Average time per consultation**											
Average time per consultation	0.01	1.01 (0.98 to 1.04)	.41								
Average time per consultation^c^	-0.01	0.99 (0.97 to 1.01)	.30*								
**Average contact frequency** (< 10)	-0.81	0.45 (0.15 to 1.36)	.15*								
**Intention to discuss** (yes)	1.25	3.49 (1.20 to 10.15)	.02*	1.32	3.76 (1.10 to 14.83)	.04	1.29	3.65 (1.01 to 13.19)	.05	0.93	2.54
**Awareness**	0.06	1.06 (0.92 to 1.22)	.42								
**Attitude**	0.15	1.16 (1.03 to 1.31)	.01*								
**Self-efficacy**	0.10	1.11 (1.02 to 1.20)	.01*	0.09	1.09 (1.00 to 1.21)	.07	0.09	1.09 (0.99 to 1.20)	.08	0.06	1.06
**Social influence**	0.14	1.15 (1.05 to 1.27)	.004*	0.14	1.15 (1.03 to 1.31)	.02	0.14	1.15 (1.02 to 1.30)	.02	0.10	1.11
**Confidence**	0.08	1.09 (1.01 to 1.16)	.02*								
**Barriers**	-0.07	0.93 (0.86 to 1.00)	.05*								

*OR* Odds ratio, *CI* Confidence interval

^*^Predictor is included in development prediction model

^a^Internal validation of performance was estimated with bootstrapping (1000 replications)

^b^Final model with regression coefficients corrected for optimism with the shrinkage factor: 0.7033

^c^Spline coefficient of variable

The derived model explained 38.9% of the total variance (Nagelkerke R^2^). The Brier score was 0.11. The Hosmer and Lemeshow test yielded a χ28 of 11.58 and showed no statistically significant difference between predicted and measured outcomes (*P* = 0.171), suggesting that the model fitted the data well. The AUC of 0.850 (95% CI: 0.772, 0.929) showed that in 85% of the cases the model correctly discriminated participants from discussing feelings and not discussing feelings.

**Fig. 2:**
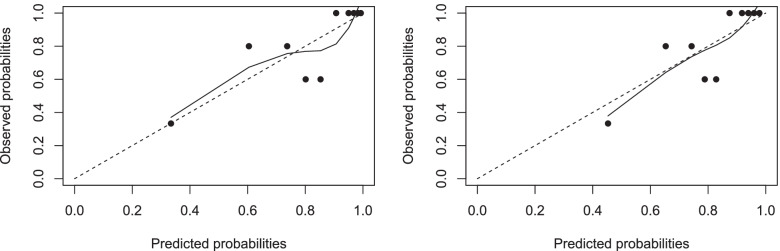
**A, B** Calibration plot original model (left) and recalibrated model after correcting for optimism (right)

**Fig. 3:**
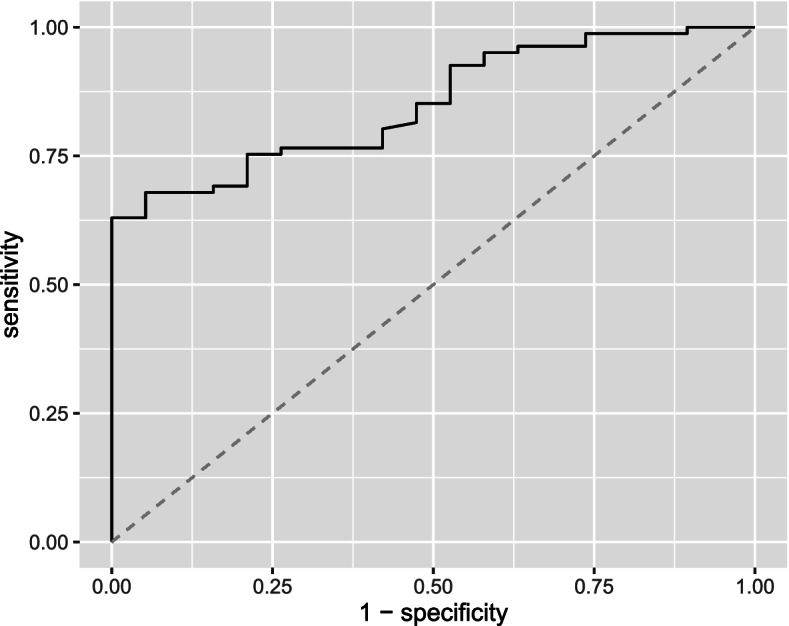
Receiver operating characteristic (ROC) curve for discussing mental health recalibrated model

Internal validation based on bootstrapping, showed that the model will discriminate less accurately in future similar participants (AUC 0.784). The developed model had overfitted regression coefficients and needed correction for optimism. The calibration slope of 0.7033, also called the shrinkage factor, was used to correct the regression coefficients for overfitting. Adjusting regression coefficients and intercept for optimism showed better agreement between observed and predicted probabilities in the calibration plots (Fig. 2A and B), and good discrimination (AUC 0.850, 95 CI: 0.772, 0.929, Fig. 3). Table 4 shows an overview of all performance measures of the original, internally validated and recalibrated model.

**Table 4:** Performance of prediction models for discussion of mental health

Performance measure	Original model	Internally validated model	Recalibrated model
R2 (Nagelkerke)	39%	25%	39%
Brier	0.11	0.14	0.11
AUC	0.85	0.78	0.85
Hosmer & Lemeshow test	χ2 = 11.58, *P* = 0.17	-	χ2 = 11.10, *P* = 0.20

## Discussion

In this study we examined factors associated with discussing depression and anxiety in visually impaired and blind adults by LVS workers. All participants believed detection of mental health is part of their job and often recognized symptoms of depression and anxiety. Many LVS workers discussed client’s feelings, but information was less often provided and only a few used a screening instrument. LVS workers that were male, had lower levels of education, did not intend to discuss mental health, experienced lower self-efficacy and lower social influence within their organization were less likely to discuss mental health.

Findings suggest that LVS workers are aware of symptoms of mental health problems. Almost all symptoms were recognized by LVS workers as part of depression or anxiety. However, symptoms were also linked to being visually impaired and similar findings are found in eye health professionals [25]. This seems a reasonable response, since some mental health symptoms, such as fatigue or decrease of social activities [21], are also specifically associated with being visually impaired [26, 27]. However, this might complicate attribution of symptoms and could result in overlooking them. Training and standardized use of a screening instrument could help LVS workers to accurately identify depression and anxiety in their clients. While different screening instruments can be used for this purpose, the Patient Health Questionnaire (PHQ)-4 would be a good choice, since it is a short questionnaire to screen for depression and anxiety [28], can be used by healthcare providers without training in psychiatry [3], and is feasible for use in LVS organizations [29].

LVS workers often addressed mental health problems by reporting or discussing concerns or client’s feelings. Discussing client’s feelings seems an important first step in management of depression and anxiety, since clients get the opportunity to open up about possible mental health problems. Still, one in five LVS workers often did not discuss suspected mental health problems, and might not meet the needs of visually impaired and blind adults to receive information about mental health problems and support options from their healthcare providers [11]. Only a quarter of the LVS workers often provided verbal information about mental health problems, and almost none of them often provided written information, which can be adapted for their clients by using e.g. Braille, large print. Providing information about depression or anxiety can improve the mental health literacy of clients, resulting in a well-informed client who can make health decisions [30], such as following-up on referrals to general practitioners and psychologists. Encouraging LVS workers to address client’s mental health could be strengthened by teaching additional depression and anxiety management strategies to improve quality of their mental health support.

Increasing LVS workers’ intention to discuss mental health, their self-efficacy and social support in their workspace seems to increase their likelihood of discussing mental health with clients. This might be the result of mental health not being the main focus of care in LVS organizations, and healthcare providers experiencing barriers in managing mental health problems, such as lack of knowledge, clients’ reluctance to discuss mental health and clients not expecting healthcare providers to discuss mental health problems [13–15]. Higher levels of self-efficacy might overcome these barriers, since LVS workers may then feel more competent to discuss their concerns, even in reluctant or denying clients. Low self-efficacy may be caused by lack of experience in depression and anxiety management. LVS workers might fear their incompetence resulting in discomfort in clients or even deteriorating client’s mental health; barriers previously reported by eye care practitioners [14]. Healthcare providers might report a need of proper training in managing mental health [14, 15, 25], while they do not encounter such situations on a daily basis, and self-efficacy can be enhanced by experiencing successful outcomes in discussing mental health [31].

Results also suggest that LVS workers are encouraged to provide non-vision related care by perceived social influence within their organization. Knowing that colleagues are discussing mental health as well, might reduce feelings of inappropriateness [32], and might encourage LVS workers to ask their colleagues for help. The effect of social influence on healthcare providers’ behavior is illustrated in social norm interventions. Within these interventions healthcare providers are exposed to values, beliefs, attitudes or behaviors of other healthcare providers and it demonstrates improvement in their clinical behavior [33]. According to the I-Change model, LVS workers’ intention to discuss mental health problems is affected by their perceived social influence and self-efficacy [17]. LVS workers that intend to discuss their concerns about symptoms of depression or anxiety might discuss client’s feelings and invite clients to discuss mental health problems as an opportunity to subsequently express their own concerns. Altogether, an organization where discussing mental health is part of their care, and training in depression and anxiety management is provided, seems to create a work environment where LVS workers can overcome perceived barriers and address mental health problems more often.

### Implications for clinical practice

LVS organizations could facilitate LVS workers to discuss mental health by creating a working environment that also focusses on client’s mental health. They should incorporate detection and support for mental health problems into their care policy and regulations, introduce screening as a standard procedure, employ psychologists, and implement evidence-based treatment for depression and anxiety, such as stepped-care [8]. Especially in health care systems where referrals or access to specialists are not feasible.

Moreover, a training in discussing mental health problems could be introduced. Previous depression training in Wales and Australia showed positive results [32, 34]. Existing educational programs could be further developed by including recent insights in the client’s perspective [11], and by addressing LVS workers’ self-efficacy, perceived social influence and intention to discuss mental health. Training LVS workers in discussing mental health with clients and them experiencing successful outcomes in their own behavior enhances self-efficacy [31]. Furthermore, principles of social norm interventions could be used, including professional supervision, in which perceived social influence can be increased by improving the working environment with better teamwork and more support from within the organization [33, 35]. Improving LVS workers’ self-efficacy and perceived social influence might result in a higher intention to discuss mental health [36]. Trainers might include goal setting, a common feature of behavior change interventions [37], to help LVS workers to set goals and develop an action plan to discuss mental health. Moreover, LVS workers should be stimulated to think about specific moments when they want to discuss mental health with their clients, also called "if–then plans" to promote their intention to reach their goals [38]. Altogether, a training could consist of an e-learning to share knowledge about depression and anxiety and support options, a meeting to practice discussing mental health problems, and a session to share and discuss experiences in practice.

### Strengths and limitations

Our study has uncovered predictors in depression and anxiety management in LVS workers, while previous studies mainly focused on eye care practitioners and depression management. Findings suggest that anxiety and depression management are comparable, and previous studies on depression might be transferable to anxiety. Use of the I-Change model as a theoretical framework helped to delineate potential predictors. While we were unable to perform IRT-analyses (Additional file 2), we could rely on classical test theory and additional measures to ensure psychometric properties of the questionnaire. However, results should be interpreted with caution since these are based on cross-sectional data, and therefore it is impossible to deduce the causality between the predictors and outcome. Moreover, participants might have had more interest in mental health than non-responders, which seems to be reflected in all participants experiencing detection of mental health as part of their job. This indicates a possible risk of selection bias.

This study lacked external validation of the model, but future studies could examine generalizability of the model in other healthcare providers working with adults with VI. In-depth studies could further explore potential mechanisms between found predictors and discussion of mental health by LVS workers. For example, knowing the impact of specific client characteristics in LVS workers’ approaches, contributes to the development of specific guidelines. Moreover, it is still unclear how often mental health problems are recognized and discussed in adults with VI, what external factors (e.g. information resources and referral options) affect discussion of mental health problems, and how LVS workers can be encouraged to use other depression and anxiety management strategies, such as providing information about mental health. Other beneficial future work lies in investigating how discussions about mental health are managed by LVS workers, and subsequently client’s experiences. Future research into these subjects could help us to better understand and improve current practice.

## Conclusion

LVS workers are more likely to discuss mental health problems in clients if they intend to discuss their own suspicions, believe they can perform well, and feel supported from within their organization. LVS organizations should encourage their employees to address mental health more often, and provide them with a supportive working environment. LVS workers seem to benefit from standardized use of a screening instrument to distinguish mental health problems from symptoms of VI, and receiving training to deploy more depression and anxiety management strategies and improve clinician-patient communication. Current educational programs could be adjusted in order to improve LVS workers’ intention, self-efficacy and feelings of social support, and increase their skills to detect and discuss mental health problems in adults with VI.

## Supplementary Information

**Additional file 1.** Portable document format (.pdf); Study Questionnaire; A copy of the final questionnaire used in this study.

**Additional file 2.** Portable document format (.pdf); Psychometric assessment of measures; Details about psychometric analyses of scales in the study.

**Additional file 3.** Portable document format (.pdf); Use of depression and anxiety management strategies; An overview of participants’ responses on use of depression and anxiety management strategies.

## Data Availability

The datasets used and analyzed during the current study are available from the corresponding author on reasonable request.

## Abbreviations

AUC: Area under the curve
CI: Confidence interval
LVS: Low vision service
SD: Standard deviation
VI: Vision impairment
