# Supplementary material for: Improved intention, self-efficacy and social influence in the workspace may help low vision service workers to discuss depression and anxiety with visually impaired and blind adults
Source: BMC Health Serv Res. 2022 Apr 21;22:528. doi: 10.1186/s12913-022-07944-0 (PMC9027071; doi:10.1186/s12913-022-07944-0)
Supplement: Supplementary file 1 — Additional file 1. Portable document format (.pdf); Study Questionnaire; A copy of the final questionnaire used in this study. [file 12913_2022_7944_MOESM1_ESM.pdf]

Additional file 1. Study Questionnaire

Part 1: Personal information

1. What is your age? _____ years
2. What is your gender?
   □ Male □ Female
3. What is your highest educational level?
   □ Primary school □ Vocational training □ Higher education □ University
4. What is your professional background?
   □ Counsellor □ Occupational therapist □ Social worker
   □ Professional that performs eligibility assessments □ Other (please specify) ______
5. How many years have you been working in low vision services? _____ years
6. How many clients do you see per week (on average)? ____
7. How much time do you have for a consultation with a client (on average)? ____ minutes
8. How many times are you in contact with a client whilst supporting them (on average)?
   □ 1 □ 2 – 10 □ > 10

9. Do you think detecting symptoms of depression and anxiety is part of your care for clients with vision impairment?
   □ Yes □ No

Part 2: Current practice

We are interested in how you currently deal with identifying symptoms of depression and anxiety in your clients.

1. If you suspect depression or anxiety in a client you are seeing next week, do you intend to discuss this with the client?
   □ Definitely not □ Probably not □ Maybe □ Probably □ Definitely

2. If you suspect depression or anxiety in a client, how likely are you to…

	Never	Rarely	Sometimes	Often
Discuss my concerns with client	1	2	3	4
Discuss client's feelings	1	2	3	4
Normalize client's feelings	1	2	3	4
Provide verbal information about depression or anxiety	1	2	3	4
Provide written information about depression or anxiety	1	2	3	4
Discuss my concerns with client's relatives (if possible)	1	2	3	4
Avoid discussing client's feelings	1	2	3	4
Use a questionnaire to measure depression/anxiety	1	2	3	4
Report concerns in client's medical file	1	2	3	4
Discuss concerns with a colleague	1	2	3	4
Provide support	1	2	3	4
Discuss referral options	1	2	3	4

3. Whenever you are unable to provide sufficient support to a client with symptoms of depression or anxiety, how likely are you to …

	**Never**	**Rarely**	**Sometimes**	**Often**
Refer to a support group	0	1	2	3
Refer to client's general practitioner	0	1	2	3
Refer to an internal or external social worker	0	1	2	3
Refer to an internal or external psychologist	0	1	2	3
Refer to a mental health care organization	0	1	2	3
Refer to another healthcare provider. Please explain ___	0	1	2	3

Part 3: Symptoms and opinion about depression and anxiety

1. What do you think the following symptoms could be related to? Select by ticking a box for "Vision impairment", "Depression", "Both" or "Neither".

	**Vision impairment**	**Depression**	**Both**	**Neither**
Depressed mood	1	2	3	4
Loss of interest in activities	1	2	3	4
Sudden weight loss or increase	1	2	3	4
Decrease or increase of appetite	1	2	3	4
Sleeping problems	1	2	3	4
Fatigue	1	2	3	4
Worthlessness	1	2	3	4
Concentration problems	1	2	3	4
Recurring thoughts about death	1	2	3	4
Decreased interest in sex	1	2	3	4
Hopelessness	1	2	3	4
Irritation	1	2	3	4
Feelings of guilt	1	2	3	4
Physical symptoms, e.g. heavy limbs, headaches, back pain and muscle pain	1	2	3	4

2. What do you think the following symptoms could be related to? Select by ticking a box for "Vision impairment", "Anxiety", "Both" or "Neither".

	**Vision impairment**	**Anxiety**	**Both**	**Neither**
Restlessness	1	2	3	4
Fatigue	1	2	3	4
Concentration problems	1	2	3	4
Irritability	1	2	3	4
Sleeping problems	1	2	3	4
Worrying about the future	1	2	3	4
Ruminating	1	2	3	4
Helplessness	1	2	3	4
Loss of control	1	2	3	4
Avoiding (social) situations	1	2	3	4
Staying at home	1	2	3	4
Uncomfortable being alone	1	2	3	4
Muscle tensions	1	2	3	4
Physical symptoms, e.g. shaking, hyperventilation and palpitations	1	2	3	4

3. We are interested in your opinion about symptoms of depression and anxiety in people with vision impairment. Please indicate to what extent you disagree or agree with the following statements by ticking the appropriate box. **Please note:** In all statements you are specifically asked about symptoms of depression **or** anxiety.

	**Strongly disagree**	**Slightly disagree**	**Slightly agree**	**Strongly disagree**
People with vision impairment are more likely to suffer symptoms of **depression** than those without	1	2	3	4
People with vision impairment are more likely to suffer symptoms of **anxiety** than those without	1	2	3	4
In most cases there is little that can be done to help someone with vision impairment with symptoms of **depression**	1	2	3	4
In most cases there is little that can be done to help someone with a vision impairment with symptoms of **anxiety**	1	2	3	4
Both medications and psychological treatments can be effective at treating symptoms of **depression**	1	2	3	4
Both medications and psychological treatments can be effective at treating symptoms of **anxiety**	1	2	3	4
Symptoms of **depression** often reoccur in people with vision impairment	1	2	3	4
Symptoms of **anxiety** often reoccur in people with vision impairment	1	2	3	4
In people with vision impairment an additional disability increases the risk of symptoms of **depression**	1	2	3	4
In people with vision impairment an additional disability increases the risk of symptoms of **anxiety**	1	2	3	4
Symptoms of **depression** are a normal response to vision loss	1	2	3	4
Symptoms of **anxiety** are a normal response to vision loss	1	2	3	4
All people with vision loss will experience symptoms of **depression** at some point	1	2	3	4
All people with vision loss will experience symptoms of **anxiety** at some point	1	2	3	4
Symptoms of **depression** are an additional cause of disability in people with vision impairment	1	2	3	4
Symptoms of **anxiety** are an additional cause of disability in people with vision impairment	1	2	3	4
People with vision impairment are more likely to experience reoccurring symptoms of **depression** than those without	1	2	3	4
People with vision impairment are more likely to experience reoccurring symptoms of **anxiety** than those without	1	2	3	4

People with symptoms of **depression** are at increased risk to develop an actual **depressive disorder**	1	2	3	4
People with symptoms of **anxiety** are at increased risk to develop an actual **anxiety disorder**	1	2	3	4
A vision impaired person with symptoms of **depression** will feel better over time	1	2	3	4
A vision impaired person with symptoms of **anxiety** will feel better over time	1	2	3	4
People may actually experience symptoms of **depression** even though they do not report feeling unhappy	1	2	3	4
People may actually experience symptoms of **anxiety** without visible symptoms	1	2	3	4
Symptoms of **depression** are a normal reaction to changes of old age	1	2	3	4
Symptoms of **anxiety** are a normal reaction to changes of old age	1	2	3	4

Part 4: Recognizing and discussing symptoms of depression and anxiety in clients with vision impairment

1. We are interested in your opinion about recognizing and discussing depression and anxiety symptoms. Please indicate to what extent you disagree or agree with the following statements by ticking the appropriate box. **Whenever I discuss symptoms of depression or anxiety with a client, I …**

	**Strongly disagree**	**Slightly disagree**	**Slightly agree**	**Strongly disagree**
provide clients the opportunity to discuss	1	2	3	4
can refer clients to another healthcare provider	1	2	3	4
improve my support to clients	1	2	3	4
show that I care about clients' mental health	1	2	3	4
prevent clients from getting more (serious) complaints	1	2	3	4
am a better healthcare provider	1	2	3	4
improve the relationship with clients	1	2	3	4
harm the relationship with clients	1	2	3	4
enhance the rehabilitation process (i.e. learn to cope with the vision impairment)	1	2	3	4
hinder the rehabilitation process (i.e. learn to cope with the vision impairment)	1	2	3	4
am afraid clients perceive it as a violation of their privacy	1	2	3	4
am afraid that clients will reject, because they want me to help with the vision impairment	1	2	3	4

2. Please indicate to what extent you disagree or agree with the following statements by ticking the appropriate box.

	**Strongly disagree**	**Slightly disagree**	**Slightly agree**	**Strongly disagree**
If a client tells you that s/he experiences symptoms of depression or anxiety, it is best to leave them alone. Talking about it might make things worse	1	2	3	4
Only clients can resolve symptoms of depression and anxiety	1	2	3	4
If I discuss symptoms of depression and anxiety, I can help clients	1	2	3	4
Thinking along about possible solutions for symptoms of depression and anxiety might help clients	1	2	3	4

3. We are interested in how your environment deals with the identification of depression and anxiety symptoms. Please indicate to what extent you disagree or agree with the following statements by ticking the appropriate box.

	**Strongly disagree**	**Slightly disagree**	**Slightly agree**	**Strongly disagree**
**My manager** does not believe that detecting symptoms of depression and anxiety is part of my role at work	1	2	3	4
**Psychologists that I work with** do not believe that detecting symptoms of depression and anxiety is part of my role at work	1	2	3	4
**Colleagues in the same profession** do not believe that detecting symptoms of depression and anxiety is part of my role at work	1	2	3	4
**My manager** is reluctant to listen to my concerns that a patient might experience symptoms of depression or anxiety	1	2	3	4
**Psychologists that I work with** are reluctant to listen to my concerns that a patient might experience symptoms of depression or anxiety	1	2	3	4
**Colleagues in the same profession** are reluctant to listen to my concerns that a patient might experience symptoms of depression or anxiety	1	2	3	4
**Colleagues in the same profession** discuss symptoms of depression and anxiety with clients	1	2	3	4
**My manager** encourages me to discuss symptoms of depression and anxiety with clients	1	2	3	4
**Psychologists I work with** encourage me to discuss symptoms of depression and anxiety with clients	1	2	3	4
**Colleagues in the same profession** encourage me to discuss symptoms of depression and anxiety with clients	1	2	3	4

**My manager** provides support in how I can discuss symptoms of depression and anxiety with clients	1	2	3	4
**Psychologists I work with** provide support in how I can discuss symptoms of depression and anxiety with clients	1	2	3	4
**Colleagues in the same profession** provide support in how I can discuss symptoms of depression and anxiety with clients	1	2	3	4
**My private environment** think it is normal to talk about symptoms of depression and anxiety	1	2	3	4

Part 5: Confidence in working with clients with vision impairment and symptoms of depression or anxiety

We are interested in how you feel about working with clients with a vision impairment and symptoms of depression or anxiety.

1. Rate how difficult or easy it is for you to discuss suspected symptoms of depression and anxiety in the following situations:

	Very difficult	Slightly difficult	Slightly easy	Very easy
Suspicions are weak	1	2	3	4
Lacking time to discuss my suspicions	1	2	3	4
Client seems reluctant to discuss	1	2	3	4
Client has a low level of education	1	2	3	4
Client has a high level of education	1	2	3	4
Client has a cognitive and/or intellectual disability	1	2	3	4
Client has physical comorbidities (such as diabetes, cancer, heart- or vascular disease)	1	2	3	4
Client has psychiatric comorbidities (such as personality disorder or autism spectrum disorder)	1	2	3	4
Client experiences difficulties with the Dutch language	1	2	3	4
Client has another cultural background	1	2	3	4
Conversation is by telephone	1	2	3	4
Conversation is face-to-face	1	2	3	4
Talking to client for the first time	1	2	3	4
Knowing client for a longer period	1	2	3	4

2. Please indicate how confident you feel in working with clients with vision impairment and symptoms of depression or anxiety by ticking the appropriate box. **Please note:** for most statements, you are specifically asked about symptoms of depression **or** anxiety.

	**Not confident at all**	**Slightly confident**	**Overwegend zelfverzekerd**	**Very confident**
In asking patients with vision impairment about their feelings or mood, I feel …	1	2	3	4
In listening to patients with vision impairment talk about their feelings or mood, I feel …	1	2	3	4
In knowing if a patient might have symptoms of **depression** or is just dissatisfied with their current situation, I feel …	1	2	3	4
In knowing if a patient might have symptoms of **anxiety** or is just insecure about the current situation, I feel…	1	2	3	4
In empathizing with the client's situation, I feel…	1	2	3	4
In being able to recognize that a patient with vision impairment might experience symptoms of **depression**, I feel ...	1	2	3	4
In being able to recognize that a patient with vision impairment might experience symptoms of **anxiety**, I feel ...	1	2	3	4
In knowing which signs to look for to tell if a patient with vision impairment might experience symptoms of **depression**, I feel ...	1	2	3	4
In knowing which signs to look for to tell if a patient with vision impairment might experience symptoms of **anxiety**, I feel ...	1	2	3	4
In deciding what to do if I suspect symptoms of **depression** in a client, I feel …	1	2	3	4
In deciding what to do if I suspect symptoms of **anxiety** in a client, I feel …	1	2	3	4
In providing education on the link between vision impairment and symptoms of **depression**, I feel …	1	2	3	4
In providing education on the link between vision impairment and symptoms of **anxiety**, I feel …	1	2	3	4
In providing education on possible treatment strategies for symptoms of **depression**, I feel …	1	2	3	4
In providing education on possible treatment strategies for symptoms of **anxiety**, I feel …	1	2	3	4
In directing a patient to appropriate services or agencies for symptoms of **depression**, I feel …	1	2	3	4

In directing a patient to appropriate services or agencies for symptoms of **anxiety**, I feel …	1	2	3	4
In discussing my concern that a client might experience symptoms of **depression** with my manager, I feel …	1	2	3	4
In discussing my concern that a client might experience symptoms of **anxiety** with my manager, I feel …	1	2	3	4
In discussing my concern that a client might experience symptoms of **depression** with my colleagues, I feel …	1	2	3	4
In discussing my concern that a client might experience symptoms of **anxiety** with my colleagues, I feel …	1	2	3	4
In supporting clients with symptoms of **depression**, I feel …	1	2	3	4
In supporting clients with symptoms of **anxiety**, I feel …	1	2	3	4
In passing on my concerns about possible symptoms of **depression** to a patient's general practitioner, I feel …	1	2	3	4
In passing on my concerns about possible symptoms of **anxiety** to a patient's general practitioner, I feel …	1	2	3	4
In discussing my concerns about possible symptoms of **depression** with a patient's family members, I feel …	1	2	3	4
In discussing my concerns about possible symptoms of **anxiety** with a patient's family members, I feel …	1	2	3	4
Overall, in providing care for clients with symptoms of **depression**, I feel …	1	2	3	4
Overall, in providing care for clients with symptoms of **anxiety**, I feel …	1	2	3	4

Part 6: Barriers in working with clients with vision impairment and symptoms of depression or anxiety

We are interested in what might complicate identifying and discussing symptoms of depression and anxiety

1. Please indicate to what extent you disagree or agree with the following statements by ticking the appropriate box.

	Strongly disagree	Slightly disagree	Slightly agree	Strongly agree
I don't have enough time to talk with patients to tell if they might experience symptoms of depression or anxiety	1	2	3	4
My high workload makes it difficult to know if a patient might experience symptoms of depression or anxiety	1	2	3	4
I haven't received enough training to know if a patient might experience symptoms of depression or anxiety	1	2	3	4
Due to the absence of standard procedures about how to deal with symptoms of depression and anxiety clients do not always receive the best support	1	2	3	4
My limited knowledge of depression and anxiety means that patients may not always receive the best management for depression and anxiety	1	2	3	4
My poor knowledge of what to do if a client experiences symptoms of depression or anxiety means that they may not always receive the best support	1	2	3	4
Since I do not meet clients regularly, I am unable to notice changes in their mood	1	2	3	4
Symptoms of depression and anxiety are not addressed because the environment in which I work is not suitable for private discussions about emotional well-being.	1	2	3	4
Family members attending the consultation means it is difficult to have an open discussion about symptoms of depression and anxiety with clients	1	2	3	4
Clients' reluctance to discuss how they feel makes it difficult to tell if they might experience symptoms of depression or anxiety	1	2	3	4
Symptoms of depression and anxiety are not explored, because I need to protect myself from being involved with clients' emotional problems	1	2	3	4
Depression and anxiety do not receive enough attention, because my role is related to clients' eye health rather than emotional well-being	1	2	3	4
Language and/or cultural barriers make it difficult to discuss symptoms of depression and anxiety with clients	1	2	3	4

Additional problems, such as intellectual disabilities or psychiatric problems, complicates discussing symptoms of depression and anxiety. Please specify _____	1	2	3	4
Most clients are reluctant to discuss mental health problems	1	2	3	4
I lack knowledge about how to discuss symptoms of depression and anxiety with clients	1	2	3	4
A long-term relationship with clients results in missing symptoms of depression and anxiety	1	2	3	4
I avoid discussing symptoms of depression and anxiety, because I have to protect my client's boundaries	1	2	3	4
A rehabilitation process (learning how to deal with vision impairment) limits discussing symptoms of depression and anxiety with clients	1	2	3	4
