# Supplementary material for: Improved intention, self-efficacy and social influence in the workspace may help low vision service workers to discuss depression and anxiety with visually impaired and blind adults
Source: BMC Health Serv Res. 2022 Apr 21;22:528. doi: 10.1186/s12913-022-07944-0 (PMC9027071; doi:10.1186/s12913-022-07944-0)
Supplement: Supplementary file 2 — Additional file 2. Portable document format (.pdf); Psychometric assessment of measures; Details about psychometric analyses of scales in the study. [file 12913_2022_7944_MOESM2_ESM.pdf]

Additional file 2. Psychometric assessment of measures

To ensure psychometric properties of all scales, i.e. awareness, attitude, self-efficacy, social influence confidence and barriers, several psychometric analyses were performed (Table 1). To assess the scales' reliability the Cronbach's alpha was computed, which showed moderate to good reliability. Subsequently, principle component analyses were performed to assess unidimensionality for each scale by determining acceleration factors and percentage of variance accounted by the first factor. Analyses showed one acceleration factor for each scale, and the first factor of each scale accounted for at least 20% of the variance, which indicates that covariance of the items is explained by a single latent trait [1,2]. In addition, local dependence between item pairs was checked, which can occur when items have more in common than the latent trait construct [3]. Analysis showed local dependence in two pairs of items (social influence and barriers, > .80), but was accepted since this seemed to occur due to similar wording and referral to the same context [2]. In addition, Item Response Theory (IRT) was performed to estimate individual latent trait scores by fitting a Graded Response Model. Only confidence in depression and anxiety showed satisfactory fit indices. Therefore, classical test theory was used by computing sum scores for each scale.

Table 1. Reliability (Cronbach's alpha) and unidimensionality (acceleration factor and variance) for all scales

	**Items**	**Cronbach's alpha**	**Acceleration factor**	**% of variance 1st component**
**Awareness** depression	13	0.54	1	23%
**Awareness** anxiety	13	0.49	1	20%
**Attitude**	16	0.71	1	21%
**Self-efficacy**	14	0.92	1	49%
**Social influence**	14	0.81	1	32%
**Confidence** depression	13	0.94	1	59%
**Confidence** anxiety	13	0.93	1	55%
**Barriers**	19	0.82	1	26%

References

1. Edelen, M. O., & Reeve, B. B. (2007). Applying item response theory (IRT) modeling to questionnaire development, evaluation, and refinement. *Quality of Life Research, 16 Suppl 1*, 5-18, doi:10.1007/s11136-007-9198-0.
2. Reeve, B. B., Hays, R. D., Bjorner, J. B., Cook, K. F., Crane, P. K., Teresi, J. A., et al. (2007). Psychometric evaluation and calibration of health-related quality of life item banks: plans for the Patient-Reported Outcomes Measurement Information System (PROMIS). *Medical Care, 45*(5 Suppl 1), S22-31, doi:10.1097/01.mlr.0000250483.85507.04.

3. Steinberg, L., & Thissen, D. (1996). Uses of item response theory and the testlet concept in the measurement of psychopathology. *Psychological Methods, 1*(1), 81-97, doi:10.1037/1082-989X.1.1.81.
