# Supplementary material for: Improved intention, self-efficacy and social influence in the workspace may help low vision service workers to discuss depression and anxiety with visually impaired and blind adults
Source: BMC Health Serv Res. 2022 Apr 21;22:528. doi: 10.1186/s12913-022-07944-0 (PMC9027071; doi:10.1186/s12913-022-07944-0)
Supplement: Supplementary file 3 — Additional file 3. Portable document format (.pdf); Use of depression and anxiety management strategies; An overview of participants’ responses on use of depression and anxiety management strategies. [file 12913_2022_7944_MOESM3_ESM.pdf]

Additional file 3. Use of depression and anxiety management strategies

**If you suspect depression or anxiety in a client, how likely are you to…**	**Never** *n* **(%)**	**Rarely** *n* **(%)**	**Sometimes** *n* **(%)**	**Often** *n* **(%)**
Discuss my concerns with client	2 (2%)	2 (2%)	22 (22%)	74 (74%)
Discuss client's feelings	0 (0%)	1 (1%)	18 (18%)	81 (81%)
Normalize client's feelings	4 (4%)	9 (9%)	31 (31%)	56 (56%)
Provide verbal information about depression or anxiety	12 (12%)	18 (18%)	43 (43%)	27 (27%)
Provide written information about depression or anxiety	48 (48%)	33 (33%)	15 (15%)	4 (4%)
Discuss my concerns with client's relatives (if possible)	8 (8%)	32 (32%)	39 (39%)	21 (21%)
Avoid discussing client's feelings	63 (63%)	32 (32%)	5 (5%)	0 (0%)
Use a questionnaire to measure depression/anxiety	85 (85%)	6 (6%)	9 (9%)	0 (0%)
Report concerns in client's medical file	0 (0%)	1 (1%)	24 (24%)	75 (75%)
Discuss concerns with a colleague	0 (0%)	0 (0%)	22 (22%)	78 (78%)
Provide support	7 (7%)	10 (10%)	42 (42%)	41 (41%)
Discuss referral options	0 (0%)	4 (4%)	47 (47%)	49 (49%)
**Referrals**				
Support group	26 (26%)	29 (29%)	39 (39%)	6 (6%)
General practitioner	3 (3%)	5 (5%)	55 (55%)	37 (37%)
Social worker	16 (16%)	20 (20%)	25 (25%)	39 (39%)
Psychologist	2 (2%)	2 (2%)	39 (39%)	57 (57%)
Mental health care organization	28 (28%)	30 (30%)	33 (33%)	9 (9%)
Other healthcare provider	60 (60%)	12 (12%)	27 (27%)	1 (1%)
